# Structure and assembly of cargo Rubisco in two native α-carboxysomes

**DOI:** 10.1038/s41467-022-32004-w

**Published:** 2022-07-25

**Authors:** Tao Ni, Yaqi Sun, Will Burn, Monsour M. J. Al-Hazeem, Yanan Zhu, Xiulian Yu, Lu-Ning Liu, Peijun Zhang

**Affiliations:** 1 Division of Structural Biology, Wellcome Centre for Human Genetics, University of Oxford, Oxford, UK; 2 Institute of Systems, Molecular and Integrative Biology, University of Liverpool, Liverpool, UK; 3 College of Marine Life Sciences, and Frontiers Science Center for Deep Ocean Multispheres and Earth System, Ocean University of China, Qingdao, China; 4 Diamond Light Source, Harwell Science and Innovation Campus, Didcot, UK; 5 Chinese Academy of Medical Sciences Oxford Institute, University of Oxford, Oxford, UK

**Keywords:** Cryoelectron tomography, Microbiology, Rubisco

## Abstract

Carboxysomes are a family of bacterial microcompartments in cyanobacteria and chemoautotrophs. They encapsulate Ribulose 1,5-bisphosphate carboxylase/oxygenase (Rubisco) and carbonic anhydrase catalyzing carbon fixation inside a proteinaceous shell. How Rubisco complexes pack within the carboxysomes is unknown. Using cryo-electron tomography, we determine the distinct 3D organization of Rubisco inside two distant α-carboxysomes from a marine α-cyanobacterium *Cyanobium* sp. PCC 7001 where Rubiscos are organized in three concentric layers, and from a chemoautotrophic bacterium *Halothiobacillus neapolitanus* where they form intertwining spirals. We further resolve the structures of native Rubisco as well as its higher-order assembly at near-atomic resolutions by subtomogram averaging. The structures surprisingly reveal that the authentic intrinsically disordered linker protein CsoS2 interacts with Rubiscos in native carboxysomes but functions distinctively in the two α-carboxysomes. In contrast to the uniform Rubisco-CsoS2 association in the *Cyanobium* α-carboxysome, CsoS2 binds only to the Rubiscos close to the shell in the *Halo* α-carboxysome. Our findings provide critical knowledge of the assembly principles of α-carboxysomes, which may aid in the rational design and repurposing of carboxysome structures for new functions.

## Abstract (web summary)

Carboxysomes are bacterial microcompartments encapsulating Rubisco and carbonic anhydrase for carbon fixation. Here, authors determine the organization of Rubisco and its interaction with the linker protein CsoS2 within two distant α-carboxysomes.

## Introduction

Bacterial cells have evolved defined internal structures, including intracellular membranes, vesicles, and membrane-less organelles, to compartmentalize and tune metabolic reactions in space and time^1,2^. Bacterial microcompartments (BMCs) are a paradigm of metabolic organelles composed purely of proteins and are widespread across the bacterial kingdom^3,4^. By sequestering key enzymes and pathways from the bacterial cytoplasm to enhance catalytic performance and reduce toxicity or unwanted side reactions, BMCs play vital roles in autotrophic CO_2_ fixation and catabolic processes^5,6^.

The first structurally discovered BMCs were carboxysomes, which serve as the central CO_2_-fixing organelles in all identified cyanobacteria and many chemoautotrophs^7–9^. The carboxysome encapsulates carbonic anhydrase and the primary CO_2_-fixing enzyme, ribulose-1,5-bisphosphate carboxylase oxygenase (Rubisco), within a protein shell that structurally resembles a virus capsid^10^. Rubisco is among the most abundant components of carboxysomes^11,12^. It is generally understood that Rubiscos are densely encapsulated within the carboxysome to overcome its slow turnover rate and off-pathway oxygen reaction. How Rubisco enzymes are organized within the carboxysome to conduct efficient carboxylation, however, has been a long-standing question.

The two lineages of carboxysomes, α- and β-carboxysomes, differ in the forms of Rubisco and their structural protein composition. It was shown that the internal organization of β-carboxysomes from freshwater β-cyanobacteria is highly packed with paracrystalline arrays of Rubisco^13,14^. This packaging, mediated by the scaffolding protein CcmM, results in the formation of a liquid-like condensate^15^, which subsequently triggers shell encapsulation and eventually construction of a full β-carboxysome^16,17^. In contrast to the β-carboxysome, the assembly process of the α-carboxysome is enigmatic. The α-carboxysome components are encoded by genes mainly in a *cso* operon in the genome^18^. The shell is constructed by CsoS1 hexameric proteins and CsoS4 pentamers. The highly conserved but intrinsically disordered protein CsoS2 was shown to function as a linker bridging the shell and the cargo Rubisco in vitro; the N-terminus of CsoS2 binds Rubisco^19^ while the C-terminus of CsoS2 is presumed to interact with shell proteins^20,21^. Previous cryo-electron tomography (cryoET) analysis of α-carboxysome has been limited to low (~40 Å) resolution^22–25^, thus Rubisco structure and its assembly within the intact α-carboxysome and biogenesis of α-carboxysomes remain unclear.

Empowered by recent advances in cryoET and subtomogram averaging (STA)^26^, we resolve the structures of Rubisco and its higher-order assembly within two distantly related native α-carboxysomes from a marine α-cyanobacterium *Cyanobium* sp. PCC 7001 (*Cyanobium*) and a chemoautotrophic bacterium *Halothiobacillus neapolitanus* (*Halo*) at near-atomic resolutions using emClarity^27,28^. The resulting structures reveal the intrinsically disordered CsoS2 bound to Rubiscos in native carboxysomes. We further determine the distinct 3D organization of Rubiscos in these two α-carboxysomes, within which CsoS2 interacts preferentially with Rubisco close to the shell in *Halo* carboxysomes, but uniformly in *Cyanobium* carboxysomes.

## Results

### Structure and assembly of Rubisco in *Cyanobium* carboxysomes

We isolated native α-carboxysomes from *Cyanobium* to high sample homogeneity (Supplementary Fig. 1a). Negative stained EM images of the isolated *Cyanobium* α-carboxysomes show 98% of particles were intact (Supplementary Fig. 1b). Cryo-EM images show that *Cyanobium* α-carboxysomes have a relatively regular shape (Supplementary Fig. 2a), which prompted us to attempt its structural determination using single particle cryoEM (SPA). However, 2D class averages indicate the structural variation of *Cyanobium* carboxysomes (Supplementary Fig. 2b). Further 3D classification only yielded a low-resolution map from a subset of 2D classes (32%) without applying symmetry, which shows a polyhedron with 20 faces and 12 vertices but deviated from a canonical icosahedron (Supplementary Fig. 2c). The limited resolution does not allow confident assignment of individual Rubiscos into density map. Interestingly, Rubisco densities are arranged in three concentric layers which are separated by 11 nm (Supplementary Fig. 2d). The individual Rubiscos, however, were not resolved. This variable morphology of *Cyanobium* carboxysomes is further confirmed by cryoET (Supplementary Fig. 2e).

**Fig. 1:**
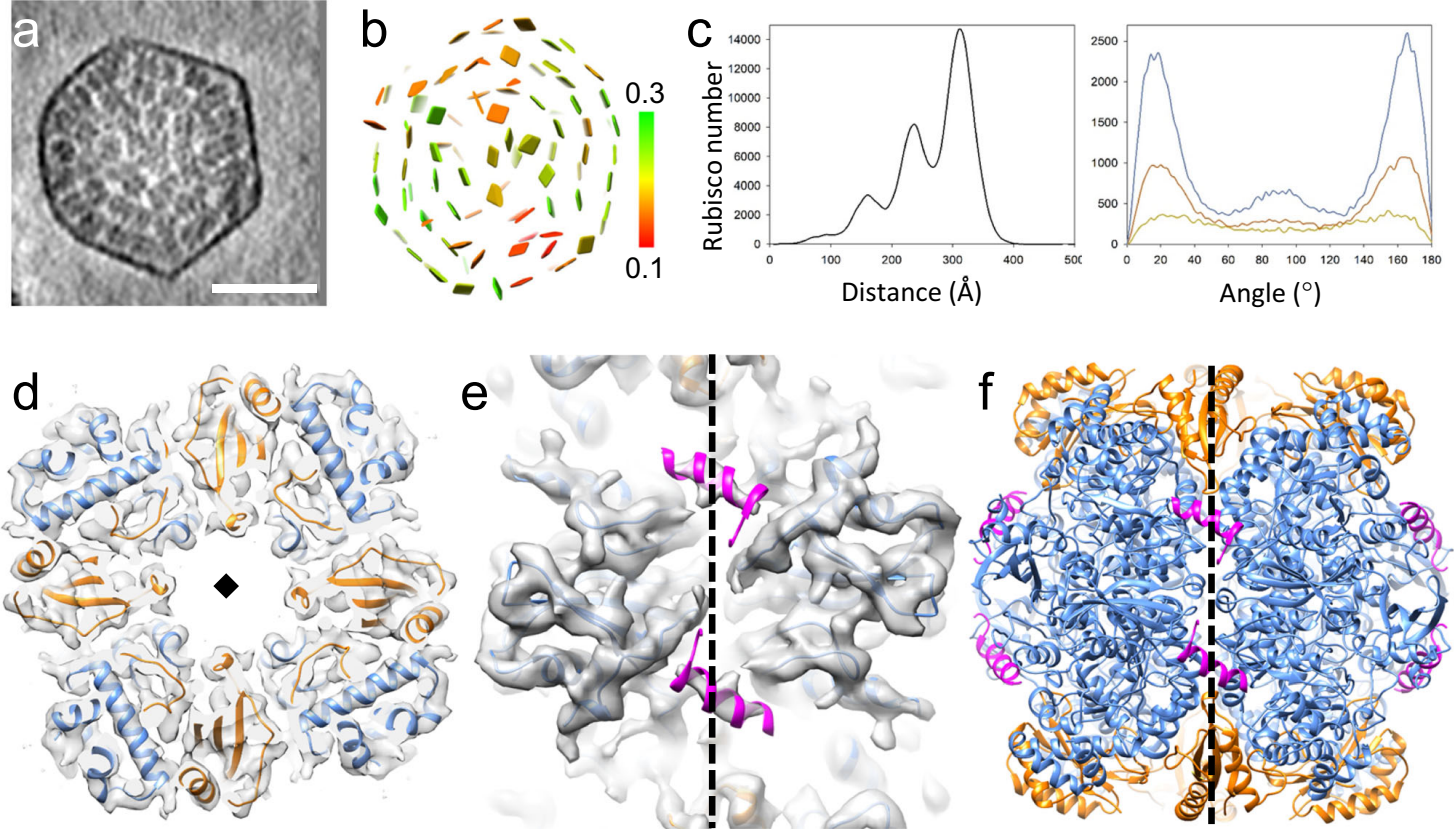
Structure and organization of Rubisco within native *Cyanobium* carboxysomes. **a** A tomograms slice (26.8 A thickness) of a *Cyanobium* carboxysome (from *n* = 137). **b** The position and orientation of individual Rubisco mapped back to the tomogram of carboxysome, shown as a square plate perpendicular to the fourfold symmetry axis of Rubisco and colored according to the cross-correlation values (0.1–0.3, red to blue) between individual Rubisco and the STA map. **c** Distributions of radial distance (measured from the center, left) and angle (measure from radial direction, right) of Rubisco in *Cyanobium* carboxysomes. **d** Cryo-ET STA structure of the Rubisco in *Cyanobium* carboxysomes at 3.8 Å resolution, overlapped with AlphaFold2 predicted atomic model, shown in a top view. CbbL and CbbS are colored in blue and gold, respectively. **e** The CsoS2 N-terminal peptide density was resolved and modeled in magenta, shown in a side view. **f** The overall atomic model of *Cyanobium* Rubisco along with the CsoS2 N-terminal peptide. The diamond and dashed lines indicate the fourfold axis. Scale bars, 50 nm. Source data are provided as a Source Data file.

To determine the structure and organization of Rubisco within native carboxysomes, we performed cryo-ET STA using emClarity^27,28^. The individual Rubisco can be readily delineated in the raw tomograms (Fig. 1a, Supplementary Movie 1). Template matching and mapping back the position and orientation of individual Rubiscos to the original tomograms revealed that Rubiscos are arranged in three concentric layers where each Rubisco is oriented with its fourfold axis along the radial direction (Fig. 1b, c). The radial distances of the three layers are peaked at 208, 308, and 413 Å (Fig. 1c left), respectively, with an angle of ~15° from the radial axis (Fig. 1c right).

We further determined the structure of Rubisco within native *Cyanobium* α-carboxysomes at an unprecedented 3.8 Å resolution by STA (Fig. 1d, Supplementary Fig. 3a, Supplementary Movie 2). Since there is no atomic model available for the *Cyanobium* Rubisco, we built an MDFF model based on alphafold2 prediction^29^ (Fig. 1f). The overall structure of *Cyanobium* Rubisco hexadecamer is very similar to its homologs, with an RMSD of 0.86 Å between this and the *Halo* Rubisco crystal structure (PDB 1SVD). We did not identify substrate density as revealed in Rubiscos from broken *Cyanobium* carboxysomes during sample preparation^30^. However, we observed an additional density that is not part of Rubisco (Fig. 1e). This density matches very well with the CsoS2 N-terminus helical peptide (Fig. 1e, magenta), as observed in the crystal structure of *Halo* Rubisco in a complex with a synthetic CsoS2 N-peptide (PDB 6UEW)^19^. The density is unlikely from CsoSCA, since CsoSCA is inserted deeply into the cleft between two CbbL, underneath the CsoS2 binding site^31^, and the occupancy of CsoSCA on Rubisco is too low (1.6%, the ratio of CsoSCA: Rubisco is 58:447), to contribute strongly to the subtomogram average^12^. The intrinsically disordered but highly conserved CsoS2 is thought to serve as the scaffolding protein connecting the carboxysome shell using its C-terminal region to Rubisco through its N-terminus^19,20^. The CsoS2 N-terminus contains four repeating segments that have similar alpha-helices, potentially all interacting with Rubisco (Supplementary Fig. 4)^19^. It is yet unclear which segment(s) is bound to the native Rubisco, as the amino acid side chains are not resolved in the subtomogram averaged map. The ratio of Rubisco (CbbL_8_/CbbS_8_) to CsoS2 is roughly 1:1.2 in a native *Halo* carboxysome^12^ and 1:0.6 in a native *Cyanobium* carboxysome (Supplementary Fig. 1a). There are eight negatively charged surface areas in the Rubisco (Supplementary Fig. 5) that provide potential binding sites for CsoS2 N-terminal segments consisting of multiple conserved Arginine and Lysine residues (Supplementary Fig. 4). Therefore, it is conceivable that these four segments from a single CsoS2 work as a scaffold wrapping around Rubisco to account for the observed eight helical densities in the STA map with about half occupancy. This scaffolding function may regulate the unique orientation of Rubisco in all three layers. It is also possible that segments of a single CsoS2 could interact with multiple Rubiscos, acting as a crosslinker.

Since CsoS2 is tethered to the shell through its C-terminus, a key question is whether CsoS2 interacts preferentially with Rubiscos close to the shell. To address this question, we obtained the subtomogram averages of Rubiscos from three different layers separately. Intriguingly, all three maps display the density corresponding to the CsoS2 N-terminal peptide (Supplementary Fig. 6), indicating its essential role in encapsulating and packaging Rubisco throughout the *Cyanobium* α-carboxysome lumen (Supplementary Movie 3).

### Structure and assembly of Rubisco in *Halo* carboxysomes

**Fig. 2:**
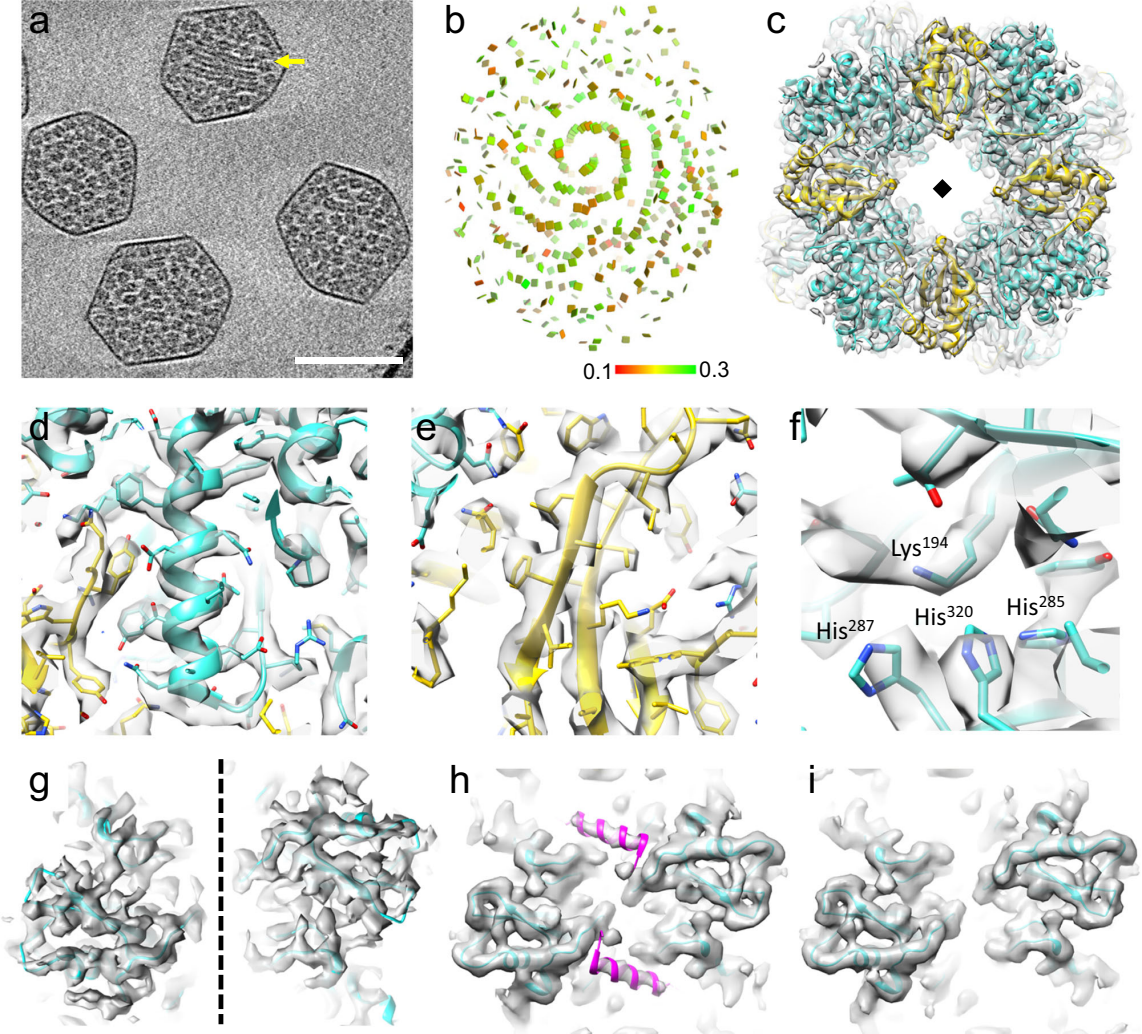
Structure and organization of Rubisco within native *Halo* carboxysomes. **a** A tomogram slice (33.5 A thickness) containing four *Halo* α-carboxysomes (from *n* = 60). Strings of Rubisco are marked by the yellow arrow. **b** The position and orientation of individual Rubisco mapped back to the tomogram of carboxysome, shown in a square plate as in Fig. 1b. **c** Cryo-ET STA structure of Rubisco at 3.3 Å resolution, overlapped with the real-space refined atomic model. CbbL and CbbS are colored in cyan and yellow, respectively. **d**, **e** Details of Rubisco density map and the atomic model are shown with side chains for CbbL (**d**) and CbbS (**e**). **f** The Rubisco catalytic site, comprising of Lys194, His287, His320, and His 285. **g**–**i** Cryo-ET STA structures of Rubiscos overlapped with the refined atomic model, viewed from the side, with data from all Rubiscos (**g**), Rubiscos close to the shell (**h**), and Rubiscos within 300 Å from the center (**i**). The CsoS2 N-terminal peptide density was resolved and modeled in magenta in (**h**). The diamond and dashed lines indicate the fourfold axis. Scale bar, 100 nm.

To understand how Rubiscos are organized in different α-carboxysomes and whether there is a conserved architecture, we analyzed a distant α-carboxysome from a chemoautotrophic bacterium, the *Halo* α-carboxysome^32^. Isolated native *Halo* α-carboxysomes were homogeneous, as indicated by SDS-PAGE (Supplementary Fig. 1a). Negative stained EM images of *Halo* α-carboxysomes show 96% of particles were intact (Supplementary Fig. 1b). Visual inspection of the tomographic reconstructions revealed that the Rubisco organization within *Halo* carboxysomes is very different from those within *Cyanobium* carboxysomes: *Halo* Rubiscos form intertwined spirals instead of concentric layers (Fig. 2a, b, yellow arrow, Supplementary Movies 4,5). Compared to the average number of 224 ± 26 Rubiscos contained in *Cyanobium* carboxysomes, there are 274 ± 72 Rubiscos in *Halo* carboxysomes (Supplementary Fig. 7a), slightly less than the stoichiometry determined by QconCAT-based quantitative mass spectrometry^12^. However, the distances between two neighbor Rubiscos in both α-carboxysomes are very similar (Supplementary Fig. 7b).

Cryo-ET STA of Rubiscos in native *Halo* carboxysomes resulted in a density map at 3.3 Å resolution, which allows a real-space refinement of the Rubisco structure (Fig. 2c–e, Supplementary Fig. 3b, Supplementary Movie 6). There is little deviation between the refined cryo-ET STA structure and the crystal structure of *Halo* Rubisco (PDB 1SVD) (RMSD of 0.35 Å). The carbamylation of Lysine 194 in the catalytic site was clearly resolved, together with three key histidine residues (Histidine 285, 287, and 320), where the positioning of these catalytic residues suggests Rubisco is at an apo state without ligand bound^33^ (Fig. 2f). Intriguingly, unlike the extra CsoS2 density identified in the *Cyanobium* Rubisco map, we observed no additional density corresponding to the CsoS2 peptide (Fig. 2g). We reasoned that this might be due to a lower overall occupancy of CsoS2 with *Halo* Rubisco and speculated that CsoS2 might have distinct associations with subpopulations of Rubisco. Therefore, we further divided Rubisco spatially and obtained STA maps of Rubisco from those close to the shell and those within 30 nm from the center, separately. Remarkably, there is a clear density corresponding to the CsoS2 helical peptide in the Rubisco complexes adjacent to the shell but is absent in the Rubiscos near the center of the carboxysome (Fig. 2h, i).

**Fig. 3:**
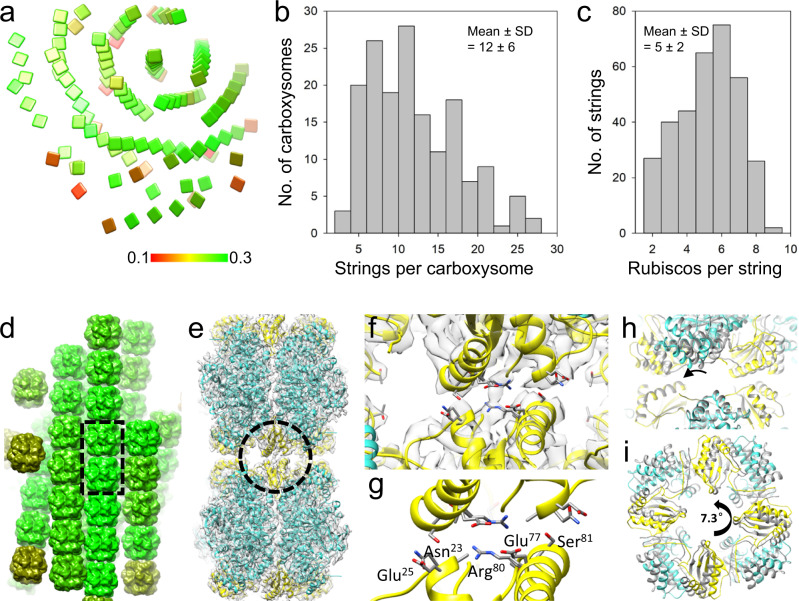
Spiral string assembly of Rubisco within native *Halo* α-carboxysomes. **a** The position and orientation of di-Rubisco mapped back to the tomogram of the *Halo* carboxysome, shown in a square plate as in Fig. 1b. **b** Histogram of Rubisco string number in *Halo* carboxysomes (*n* = 167 carboxysomes). **c** Histogram of Rubisco string length in *Halo* carboxysomes (*n* = 335 strings). **d** Rubisco subvolumes are mapped back to *Halo* carboxysome spiral strings. The dashed rectangle encloses a Rubisco dimer. **e** Cryo-ET STA structure of the Rubisco dimer at 4.1 Å resolution, overlapped with the fitted atomic model. **f**, **g** Detailed views of the Rubisco dimer interface, mediated by CbbS subunits (circled in **e**). Charged interface residues are labeled in (**g**). **h** Comparison of the dimer interface between the cryo-ET STA structure of Rubisco dimer (colored) and the crystal structure lattice contacts (PDB 1SVD, gray), viewed from the side as in (**e**). The bottom Rubisco in the dimer is aligned. **i** The overlay of the top Rubisco from cryo-ET STA dimer (colored) and crystal lattice (gray). The curved arrows indicate the rotation between the two structures. Source data are provided as a Source Data file.

*Halo* carboxysomes display various arrangements of Rubiscos, which we coarsely classified into four classes: randomly distributed (<3 Robiscos in a row), short strings (3–6 Rubiscos in a row), moderately and highly ordered (>7 Rubiscos in a row) (Supplementary Fig. 7c). In ~38% of *Halo* carboxysomes (classes of moderately and highly ordered), Rubiscos are organized in a spiral array in the middle (Figs. 2b, 3a, Supplementary Movies 4, 5), which accounts for ∼8% of total Rubiscos in these carboxysomes. The ordered spiral strings are present in nearly the entire range of populations of *Halo* carboxysomes, although the propencity is higher in those larger carboxysomes containg more Rubiscos (Supplementary Fig. 7d). The number of Rubisco strings varies among individual carboxysomes from 2 to 35 (mean ± SD = 12 ± 6, Fig. 3b), and their lengths also vary from 2 to 9 Rubiscos (mean ± SD = 5 ± 2, Fig. 3c). The Rubisco spiral array tends to localize in the center of the carboxysome and is formed by near-parallel packing of Rubisco strings: each Rubisco string is surrounded by 6 strings (Fig. 3a, Supplementary Movie 7). To understand the molecular interactions between Rubiscos in the string-like assembly, we further determined the Rubisco dimer structure at 4.1 Å resolution using cryo-ET STA and docked the atomic model of *Halo* Rubisco (Fig. 3d, e, Supplementary Fig. 3c). The Rubisco tandem dimer interface is primarily mediated by four Rubisco small subunits CbbS, providing charge interactions similar to those observed in the crystal packing (PDB 1SVD, Fig. 3f, g). However, the Rubisco tandem dimer in the string assembly is rotated about 7.3° with respect to each other, giving rise to the spiral array-like Rubisco organization within *Halo* α-carboxysomes (Fig. 3h, i, Supplementary Movie 8).

## Discussion

**Fig. 4:**
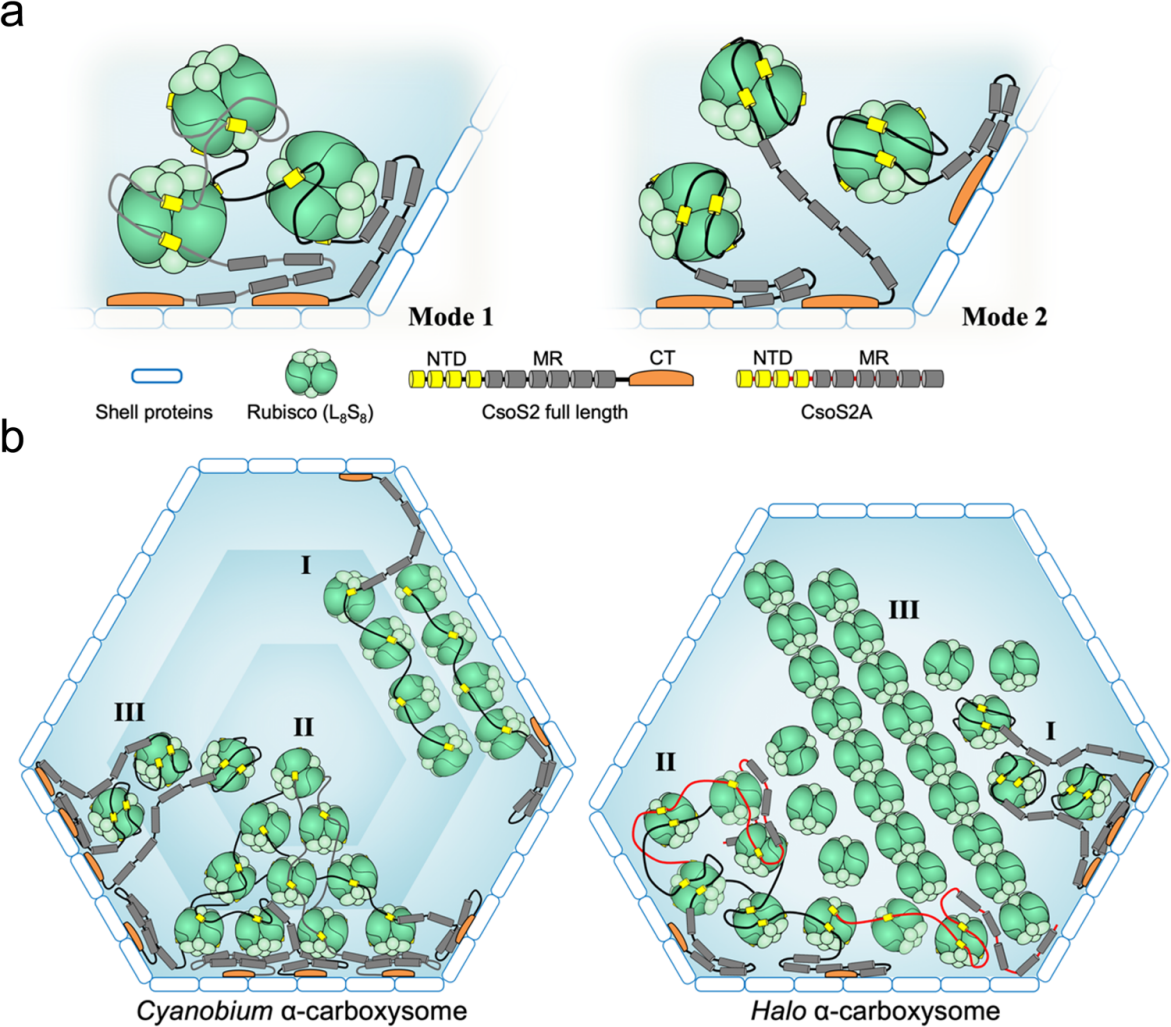
Models of CsoS2-mediated Rubisco assembly within α-carboxysomes. **a** Models of CsoS2 and Rubisco interaction within α-carboxysomes. There could be a crosslinking assembly: each N-Terminal Domain (NTD) of CsoS2 interacts with multiple Rubiscos (up to four), and each Rubisco binds multiple CsoS2 (left, Mode 1). Alternatively, there could be a pairwise assembly: each NTD of CsoS2 interacts with a single Rubisco where four highly conserved charged segments (yellow) occupy four out of eight potential binding sites on Rubisco (right, Mode 2). MR (gray), Middle Region of CsoS2 containing repeat motifs with unknown functions. CT (orange), C-terminus of CsoS2 that binds to the shell; (**b**) Models of CsoS2-mediated Rubisco packaging within C*yanobium* (left) and *Halo* (right) α-carboxysomes. Rubiscos are organized in three layers in the *Cyanobium* α-carboxysome. CsoS2, anchored to the carboxysome shell via its CT, may connect multiple Rubiscos within the same layer (I), or multiple Rubiscos across three different layers (II), or wrap around individual Rubisco (III)_,_ through its N-terminal charged fragments (yellow). In the *Halo* α-carboxysome, CsoS2 has two isoforms, the full-length CsoS2 and the C-terminal truncated isoform termed CsoS2A. Full-length CsoS2 assists in organizing Rubisco complexes close to the shell (I), perhaps together with CsoS2A (in red, II), while higher-order spiral string assembly of Rubiscos in the inner core is mediated by CbbS and does not require CsoS2 (III).

Unraveling the assembly mechanism of carboxysomes is key for understanding the biosynthesis and functions of metabolic organelles in prokaryotes and repurposing carboxysomes in diverse biotechnological applications using synthetic biology. Our cryo-ET structures capture directly the native CsoS2-Rubisco interactions within α-carboxysomes. Two possible modes of CsoS2 and Rubisco interaction can be envisaged (Fig. 4a). In the crosslinking assembly mode, each N-terminal domain of CsoS2 interacts with multiple Rubiscos (up to four), and each Rubisco binds multiple CsoS2 to form an inter-connected Rubisco network (Mode 1). In the pairwise assembly mode, each CsoS2 interacts with a single Rubisco, where four highly conserved charged segments wrap around and occupy four out of eight potential binding sites on Rubisco (Mode 2). Since CsoS2 has been shown to phase separate with Rubisco  in vitro, which indicates the formation of a higher-order Rubisco-CsoS2 network, it is more likely that each N-terminal domain of CsoS2 crosslinks multiple Rubiscos in phase separation (Fig. 4a, Mode 1). How in vitro phase separation recapitulates the Rubisco packaging within authentic carboxysomes merits further investigation.

The CsoS2-Rubisco interaction, however, could result in markedly different Rubisco organizations in two α-carboxysomes. Several reasons could contribute to the difference. First, *Cyanobium* and *Halo* Rubiscos display different surface electrostatic properties, in addition to the variation of the CsoS2 N-terminal sequence, which may affect the affinity of CsoS2 binding and Rubisco assembly (Supplementary Fig. 4). Second, the *Halo* carboxysome possesses two isoforms of CsoS2, translated via programmed ribosomal frameshifting, with one full-length CsoS2 and one truncated form (CsoS2A) that lacks the C-terminal region responsible for carboxysomal shell anchoring^12,34^ (Supplementary Fig. 3). In contrast, *Cyanobium* carboxysomes only contain the full-length form of CsoS2^30^ (Supplementary Fig. 1a). Protein abundance analysis revealed that the relative CsoS2 content in *Halo* carboxysomes was twofold of that in *Cyanobium* carboxysomes at the same Rubisco level (Supplementary Fig. 1a), which might contribute to the different packing patterns observed. Based on the structural findings herein, we propose the models of Rubisco organization in these two α-carboxysomes, as illustrated in Fig. 4b. In *Cyanobium* carboxysomes, Rubiscos are organized in three concentric layers, where CsoS2, while anchored to the carboxysome shell via its C-terminus (orange), interacts with Rubisco from all three layers: it may connect multiple Rubiscos within the same layer (Mode I), or multiple Rubiscos across three different layers (Mode II), or wrap around individual Rubisco (Mode III), through its N-terminal charged fragments (yellow). In *Halo* α-carboxysomes, CsoS2 assists in organizing the Rubiscos adjacent to the shell (Mode I), or with the truncated isoform CsoS2A (in red, Mode II), while Rubiscos spiral strings in the inner core are mediated by CbbS and do not require CsoS2 (Mode III). Nevertheless, the fact that in both α-carboxysomes Rubiscos close to the shell are connected to CsoS2 strongly suggests that Rubiscos are potentially recruited and encapsulated via CsoS2 linkage for the initial assembly, differing from the inside-out biogenesis and Rubisco interior organization of β-carboxysomes^13,16^.

A recent study reported a cryo-EM SPA structure of Rubisco particles releassed from broken *Cyanobium* carboxysome^30^. The map showed an extra unknown density at the large subunit, which is absent in our cryo-ET subtomogram average of Rubiscos from native intact carboxysome. Rubisco spirals were observed in various *Halo* carboxysomes containing different copy numbers of Rubiscos (Supplementary Fig. 7c), consistent with a recent cryo-ET study^35^. The variation of Rubisco spirals in carboxysomes indicates a general equilibrium of Rubisco assembly during carboxysome assembly, which may relate to regulating carboxysome activity. Activity assays showed that native *Halo* carboxysomes exhibited a higher *K*_mRuBP_ (150–200 µM)^12,36^ than *Cyanobium* carboxysomes (80 µM)^37^. How the distinct Rubisco organizations within the two different types of α-carboxysomes are naturally established and how they determine the catalytic activities of carboxysomes remain to be addressed.

In summary, our results provide insights into the native architecture and construction principles of α-carboxysomes and offer a framework for further investigation of Rubisco assembly and functional regulation within the intact carboxysome. It also provides new approaches for studying other BMCs like β-carboxysomes and metabolosomes in their native state.

## Methods

### Purification of α-carboxysomes

The *Halothiobacillus neapolitanus* (*Halo*) strain used in this work was acquired from ATCC (The American Type Culture Collection). Cell cultivation and *Halo* α-carboxysome purification were performed as described previously^12^. Seeding cells were maintained in liquid ATCC medium 290 or on ATCC 290 1.5% agar plates and inoculated in the Vishniac and Santer medium^38^ in a 5 L fermenter (BioFlo 115, New Brunswick Scientific, US) and were kept at constant pH 7.6 through the supplement of 3 M KOH. The growth was maintained at 30 °C with agitation kept at 250–300 rpm. The air supply was set at 500 L min^−1^ for initial growth and reduced to 200 L min^−1^ 24–48 h before cell collection. Cells were pelleted by sequential centrifugation at 12,000 × *g* for 10 min, 300 × *g* for 15 min, and 12,000 × *g* for 10 min in TEMB buffer (10 mM Tris-HCl, pH 8.0, 10 mM MgCl_2_, 20 mM NaHCO_3_, 1 mM EDTA). Cells were treated by egg lysosome (at a final concentration of 0.5 mg mL^−1^) for 1 h at 30 °C and then disrupted via glass beads beating (150–212 μm glass bead, acid washed, Sigma-Aldrich, US). The lysates were further treated with 33% (v/v) B-PERII (ThermoFisher Scientific, UK) and 0.5% (v/v) IGEPAL CA-630 (Sigma-Aldrich, US). Crude carboxysome enrichment was pelleted at 48,000 × *g*, resuspended, and then loaded to a step sucrose gradient (10–60%) for a 35-min centrifugation at 105,000 × *g*. The milky layer of enriched carboxysome was harvested, and sucrose was removed by an additional round of ultracentrifugation after dilution with TEMB buffer. The final pure carboxysome pellet was resuspended in a small volume of TEMB buffer. Unless indicated otherwise, all procedures were performed at 4 °C. Protease Inhibitor Cocktail (Sigma-Aldrich, US) was added to purified carboxysomes according to manufacturer suggestions to avoid protein degradation.

*Cyanobium* sp. PCC 7001 (Pasteur Culture Collection of Cyanobacteria, PCC) cells were grown in 4 L of BG-11 medium under constant illumination at 30 °C with constant stirring and bubbling with air. *Cyanobium* α-carboxysomes were purified as described previously^30^. Cells were collected by centrifugation (6000 × *g*, 10 min) and resuspended in TEB buffer (5 mM Tris-HCl, pH 8.0, 1 mM EDTA, 20 mM NaHCO_3_) with additional 0.55 M mannitol and 60 kU lysozyme (Sigma-Aldrich, United States). Cells were then incubated overnight (20 h) with gentle shaking at 30 °C in the dark and were collected via centrifugation (6000 g, 10 min). Cells were placed on ice and resuspended in 20 mL ice-cold TEB containing an additional 5 mL 1 µm Silicone disruption beads. Cells were broken via bead beating for 8 min in 1 min intervals of vortexing, and 1 min on ice. Broken cells were separated from the beads, and the total resuspension volume was increased to 40 mL with TEB buffer containing an additional 4% IGEPAL CA-630 (Sigma-Aldrich, United States) were mixed on a rotating shaker overnight at 4 °C. Unbroken cells were pelleted via centrifugation at 3000 × *g* for 5 min, and the supernatant was centrifuged at 40,000 × *g* for 20 min. The pellet was then resuspended in 40 mL TEMB containing 4% IGEPAL CA-630 and centrifuged again at 40,000 × *g* for 20 min. The resulting pellet was then resuspended in 2 mL TEB + 10 mM MgCl_2_ (TEMB) (5 mM Tris-HCl, pH 8.0, 1 mM EDTA, 10 mM MgCl_2_, 20 mM NaHCO_3_) and centrifuged at 5000 × *g* for 5 min before loading onto a 20–60% (v/v) sucrose gradient in TEMB buffer. Gradients were then centrifuged at 105,000 × *g* for 60 min at 4 °C; the milky band at the 40–50% interface was collected, diluted in 10 mL TEMB buffer, and centrifuged again at 105,000 × *g* for 60 min. The final carboxysome pellet was then resuspended in 150 µL TEMB for the following structural and biochemical analysis.

The purified carboxysomes were stained with 3% uranyl acetate on carbon grids and were then inspected for their quality and intactness with an FEI 120 kV Tecnai G2 Spirit BioTWIN transmission electron microscope (TEM) equipped with a Gatan Rio 16 camera, as described previously^12^.

### SDS-PAGE analysis

SDS-PAGE analysis was performed following standard procedures. 10 μg purified carboxysomal proteins were loaded per well on 15% polyacrylamide gels and stained with Coomassie Brilliant Blue G-250 (ThermoFisher Scientific, UK). Relative molar ratio of CsoS2, CsoS2B, and CsoS2A abundance was determined by measuring protein band intensities calibrated to equal CbbL content using ImageJ.

### Cryo-EM SPA sample preparation and data collection

The *Cyanobium* sample was prepared by plunge freezing in ethane onto the carbon side of Lacey ultra-thin carbon 400 mesh grids (Agar Scientific) using Vitrobot with a blotting time of 3.5 s and blotting force of −15. The Grids were glow-discharged for 45 s before use. Data were acquired with the Thermofisher 300 kV Titan Krios microscope equipped with a Falcon 4 direct electron detector with a Selectris energy filter operated with 10 eV slit width in EPU. The pixel size is 1.171 Å with a total electron dose of ∼40 e^−^/Å^2^ for each movie. 13,606 frame movies were acquired in total.

### Cryo-EM SPA data processing of *Cyanobium* carboxysome

For cryo-EM SPA of the *Cyanobium* α-carboxysomes, the beam-induced motion was corrected using MotionCor2 (v1.2.6)^39^ to generate dose-weighted micrographs from all movie frames. The contrast transfer function (CTF) was estimated using Gctf (v1.06)^40^. The particle picking, 2D and 3D classification, and final refinement were conducted in Relion3.1^41^. The particles were automatically picked using 2D class averages obtained from a subset of manually picked particles. The resulting particles were extracted at bin 4 and subject to several rounds of 2D classification and 3D classification with C1 symmetry, which resulted in a relatively clean dataset (6719 from 20,982 particles). The final refinement with C1 symmetry resulted in a density map at a resolution of 38 Å, which was presented using ChimeraX (v1.3)^42^.

### Cryo-ET sample preparation and data collection

The purified *Halo* α-carboxysomes were plunge-frozen in ethane onto lacey holy carbon grids (300 mesh, Agar Scientific) using Vitrobot or Leica GP2. The grids were glow-discharged for 45 s before plunge freezing and gold fiducial beads (6 nm) were mixed with the sample before sample application to grids. The excess solution was blotted with filter paper for 3 s with a humidity of 100% and a temperature of 20 °C. The tilt-series were acquired using a ThermoFisher Titan Krios microscope operated at 300 keV, equipped with a K2 camera and Quantum energy filter in zero-loss mode with 20 eV slit width. The tilt series were collected with SerialEM (v3.8)^43^ using a dose-symmetric tilt scheme starting from 0° with a 3° tilt increment by a group of 3 and an angular range of ±60°. The accumulated dose of each tilt series was around 120 e^−^/Å^2^ with a defocus range between −2 and −5 µm. Ten raw frames at each tilt were saved for each tilt series. Details of data collection are listed in Supplementary Table 1.

### Subtomogram averaging

Tilt-series from *Halo* carboxysomes and *Cyanobium* carboxysomes were aligned with IMOD (v4.9.12)^44^ using the gold fiducials, with the aid of in-house on-the-fly processing python script (https://github.com/ffyr2w/cet_toolbox). The center of each identified gold fiducial was manually checked. Subtomogram averaging was performed using emClarity (v1.5.0.2 and v1.5.3.11)^28^. Rubisco crystal structure (PDB: 1SVD) was converted to density map at 20 Å resolution using *molmap* command in Chimera and subsequently used as the template for template matching in emClarity. Template matching was performed with 4× binned tomograms with a pixel size of 5.36 Å (hereafter bin4 tomograms) with or without ctf correction but filtered at the first zero of CTF (contrast transfer function) in emClarity. The resulting Rubisco coordinates were manually inspected to remove the false positives and the isolated Rubiscos outside carboxysomes. The Rubisco coordinates were also carefully checked against the bin4 tomograms to ensure that most of the Rubiscos inside carboxysomes are picked up. For *Halo* carboxysomes (apostate), subtomograms from the first 60 tilt series (from 165 tilt-series) were used for subtomogram averaging and alignment. The averaging and alignment were firstly performed at bin3 with a pixel size of 4.02 Å for 4 cycles, bin2 (2.68 Å pixel size) for 8 cycles, and bin1 for 4 cycles. We performed one round of tomoCPR at bin3 after bin1 alignment and repeated the alignment at bin2 and bin1, which improved the overall density map. Duplicates of subtomograms were removed during alignment. The dataset was divided into two independent subsets during the alignment for gold-standard metrics and the two subsets were combined in the final iteration, which resulted in the final resolution of 3.3 Å. C4 symmetry was applied throughout the alignment procedure, except for the final 2 rounds of alignment using D4 symmetry. *Cyanobium* carboxysome dataset were processed similarly without tomoCPR and the final density map was reconstructed using 2D tilt-series images with cisTEM within emClarity package, at a resolution of 3.8 Å.

After the consensus alignment, Rubiscos from different positions from carboxysomes were extracted and reconstructed with cisTEM, with one round of local translational searches. Rubiscos from the three concentric layers in *Cyanobium* carboxysomes were selected based on radial distance distribution (Fig. 1c). Rubiscos within 300 Å distance from the *Halo* carboxysome center were extracted and averaged to obtain a density map representing internal Rubiscos. Rubisco close to the *Halo* carboxysomes shell were identified in the following steps: the center of individual carboxysomes was manually labeled and further refined by the mean position of all the Rubiscos within the carboxysomes, and Rubiscos within 400 Å were removed to only keep the Rubisco close to the shell. Since *Halo* carboxysomes have various sizes and morphology, a further manual inspection of the remaining Rubisco coordinates was performed to remove the Rubiscos that are not along the shell.

### Identification of Rubisco string and subtomogram averaging

The Rubisco strings were obvious in the bin6 tomograms and can be identified from synthetic tomograms in which the refined subtomograms are placed back to the tomograms according to their positioning and orientations. Manual inspection was initially performed for a small dataset. We found Rubiscos in the string have their 4-fold axis along the string and most strings are organized in a similar orientation within the same carboxysome. For the large dataset, Rubisco in the string was identified by satisfying the following geometry restraints: (i) two tandem Rubisco in the string should have their 4-fold axis pointing in the same or opposite direction, due to the D4 symmetry, and (ii) the distance between the adjacent Rubiscos should be close to the diameter of Rubisco. After finding Rubiscos satisifying these two restraints, manual inspection was performed to remove the Rubiscos that do not locate in the string. Only the Rubisco strings located in the relatively large and defined spiral arrays were included for subtomogram averaging. To obtain a map focusing on the Rubisco interface, the center of alignment box was shifted to Rubisco dimer interface along string from the Rubisco center and further few rounds of alignment were performed. Quantification of Rubisco strings in carboxysomes was performed by visual inspection, after each Rubisco subtomogram was refined. Each carboxysome was classified into the following classes according to the length of Rubisco strings: (1) no ordered Rubiscos (<3); (2) short Rubisco strings (3–6); (3) intermediate ordered Rubisco strings (>7/moderate) and (4) well-ordered Rubisco strings (>7/high). Only the well-ordered Rubisco strings were included for subtomogram averaging.

### Radial and angular distributions of Rubiscos

To calculate the radial and angular distribution of Rubiscos, the center of individual carboxysomes was calculated as the average of all Rubiscos positions in the carboxysome. The distance between each refined Rubisco and the carboxysome center was calculated to generate radial distance distribution. A radial vector for each Rubisco was calculated pointing from the center of the carboxysome to each Rubisco; the angle between the radial vector and fourfold axis or Rubisco was calculated to generate radial angular distribution.

### Model building and refinement

Crystal structure (PDB 1SVD) of Rubisco was manually fit into the subtomogram averaging density map from *Halo* carboxysome and further refined in Coot (WinCoot 0.9.9.1)^45^ and Phenix.real_space_refine (Phenix v1.20)^46^. The structure of Rubisco subunits (CbbL and CbbS) from *Cyanobium* was initially predicted using AlphaFold2^29^ and rigid-body fit into the density map to generate the full structure (8CbbL and 8CbbS). Rubisco subunit sequences were listed in Supplementary Table 2. The resulting structure was manually corrected in Coot before the molecular dynamics flexible fitting using Namdinator^47^. The surface electrostatic potential was calculated using APBS plugin^48^ in PyMOL (v2.0). Calculations were performed at 0.15 M ionic strength in monovalent salt, 298.15 K. Distribution and orientation of Rubiscos were presented in Chimera (v1.16) using Place Object plugin (v2.1.0)^49^ after converting emClarity metadata to the required format. The figures were prepared in Chimera^50^ and PyMOL^51^.

### Reporting summary

Further information on research design is available in the Nature Research Reporting Summary linked to this article.

## Supplementary information

Supplementary Information

Description of Additional Supplementary Files

Supplementary Movie 1

Supplementary Movie 2

Supplementary Movie 3

Supplementary Movie 4

Supplementary Movie 5

Supplementary Movie 6

Supplementary Movie 7

Supplementary Movie 8

Reporting Summary

## Data Availability

All data needed to evaluate the conclusions in the paper are present in the paper and/or the Supplementary Information. The cryo-ET subtomogram averaging density maps and corresponding atomic models have been deposited in the EMDB and PDB, respectively. The accession codes are listed as follows: PDB 7ZC1 and EMD-14617 (*Cyanobium* Rubisco from all the carboxysomal Rubisco), EMD-14625 (*Cyanobium* Rubisco from the outer layer), EMD-14624 (*Cyanobium* Rubisco from the middle layer), EMD-14623 (*Cyanobium* Rubisco from the inner layer), PDB 7ZBT and EMD-14590 (*Halo* Rubisco inside carboxysomes), EMD-14592 (*Halo* Rubisco close to the shell), EMD-14593 (*Halo* Rubisco within 300 Å from the carboxysome center), and EMD-14589 (*Halo* Rubisco within the spiral array). Source data are provided with this paper.

## Source data

Source Data
