# Supplementary material for: Structure and assembly of cargo Rubisco in two native α-carboxysomes
Source: Nat Commun. 2022 Jul 25;13:4299. doi: 10.1038/s41467-022-32004-w (PMC9314367; doi:10.1038/s41467-022-32004-w)
Supplement: Supplementary file 1 — Supplementary Information [file 41467_2022_32004_MOESM1_ESM.pdf]

Supplementary information for

Structure and Assembly of Cargo Rubisco in Two Native α-Carboxysomes

Tao Ni, Yaqi Sun, Will Burn, Monsour M. J. Al-Hazeem, Yanan Zhu, Xiulian Yu, Lu-Ning Liu and Peijun Zhang

Supplementary Table 1. Data collection, processing and model refinement statistics

	Cyanobium *(SPA)*	*Cyanobium*	*Halo*	
		EMD-14617<br>PDB 7ZC1	EMD-14590<br>PDB 7ZBT	EMD-14589*
**Data collection**				
Magnification	105,000	64,000	105,000	
Voltage (kV)	300	300	300	
Electron dose (e–/Å^2)	40	~150	~123	
Defocus range (μm)	-0.4 to -2.4	-2.5 to -5	-1.5 to -5	
Detector	Falcon 4<br>Selectris	K3	K2	
Energy-filter (slit)	Yes, 10 ev	Yes, 20 ev	Yes, 20 ev	
Super-res mode	N.A.	Yes	Yes	
Acquisition scheme	Single particle	-60°/60°, 3°<br>dose-symmetric	-60°/60°, 3°<br>dose-symmetric	
Frame number	EER format	10	10	
Pixel size (Å)	1.171	1.34	1.34	
No. of Tilt-series/Micrographs	13606	139	157	
**Data processing**				
No. of Tilt-series/Micrographs	13606	137	60	60
Symmetry imposed	C1	D4	D4	C4
Final particle images	6719	152317	149479	11907
Map resolution (Å)	38	3.8	3.3	4.1
FSC threshold	0.5	0.143	0.143	0.143
**Refinement**				
Initial model used (PDB code)		1SVD, 6UEW	1SVD, 6UEW	1SVD
Model resolution (Å)		4.5	3.8	4.3
FSC threshold		0.5	0.5	0.5
Map sharpening *B* factor (Å^2)		-10	-50	-10
Model composition<br>Non-hydrogen atoms<br>Protein residues<br>Ligands		 34872<br>4416 	 34912<br>4416 	
B factors (Å^2)<br>Protein<br>Ligand		 161.34 	 94.77 	
R.m.s. deviations<br>Bond lengths (Å)<br>Bond angles (°)		 0.002<br>0.553	 0.008<br>0.82	
Validation<br>MolProbity score<br>Clashscore<br>Poor rotamers (%)		 2.17<br>13.79 	 1.69<br>13.90 	
Ramachandran plot<br>Favored (%)<br>Allowed (%)<br>Disallowed (%)		 95.26<br>4.56<br>0.18	 97.81<br>2.19<br>0	

* Rubisco dimer in *Halo* spiral strings.

Supplementary Table 2. Protein sequences used in this study

Cyanobium sp. PCC 7001 **Rubisco**
CbbL (Large subunit):<br>MSKKYDAGVKEYRDTYWTPDYVPLDTDLLACFKCTGQEGVPKEEVAAAVAAESSTGTWSTVWSELLVDLDFYKGRCYRI<br>EDVPGDKEAFYAFIAYPLDLFEEGSVTNVLTSLVGNVFGFKALRHLRLEDIRFPMAFIKTCPGPPNGICVERDRMNKYG<br>RPLLGCTIKPKLGLSGKNYGRVVYECLRGGLDFTKDDENINSQPFQRWQNRFEFVAEAVALAQQETGEKKGHYLNCTAA<br>TPEEMYERAEFAKELGQPIIMHDYITGGFTANTGLSKWCRKNGMLLHIHRAMHAVIDRHPKHGIHFRVLAKCLRLSGGD<br>QLHTGTVVGKLEGDRQTTLGFIDQLRESFIPEDRSRGNFFDQDWGSMPGVFAVASGGIHVWHMPALVAIFGDDSVLQFG<br>GGTHGHPWGSAAGAAANRVALEACVKARNAGREIEKESRDILMEAAKHSPELAIALETWKEIKFEFDTVDKLDVQ
CbbS (Small subunit):<br>MPFKSTVGDYQTVATLETFGFLPPMTQDEIYDQIAYIIAQGWSPLIEHVHPSRSMATYWSYWKLPFFGEKDLGVIVSEL<br>EACHRAYPDHHVRLVGYDAYTQSQGACFVVFEGR
Halothiobacillus neapolitanus **Rubisco**
CbbL (Large subunit):<br>MAVKKYSAGVKEYRQTYWMPEYTPLDSDILACFKITPQPGVDREEAAAAVAAESSTGTWTTVWTDLLTDMDYYKGRAYR<br>IEDVPGDDAAFYAFIAYPIDLFEEGSVVNVFTSLVGNVFGFKAVRGLRLEDVRFPLAYVKTCGGPPHGIQVERDKMNKY<br>GRPLLGCTIKPKLGLSAKNYGRAVYECLRGGLDFTKDDENINSQPFMRWRDRFLFVQDATETAEAQTGERKGHYLNVTA<br>PTPEEMYKRAEFAKEIGAPIIMHDYITGGFTANTGLAKWCQDNGVLLHIHRAMHAVIDRNPNHGIHFRVLTKILRLSGG<br>DHLHTGTVVGKLEGDRASTLGWIDLLRESFIPEDRSRGIFFDQDWGSMPGVFAVASGGIHVWHMPALVNIFGDDSVLQF<br>GGGTLGHPWGNAAGAAANRVALEACVEARNQGRDIEKEGKEILTAAAQHSPELKIAMETWKEIKFEFDTVDKLDTQNR
CbbS (Small subunit):<br>MAEMQDYKQSLKYETFSYLPPMNAERIRAQIKYAIAQGWSPGIEHVEVKNSMNQYWYMWKLPFFGEQNVDNVLAEIEAC<br>RSAYPTHQVKLVAYDNYAQSLGLAFVVYRGN

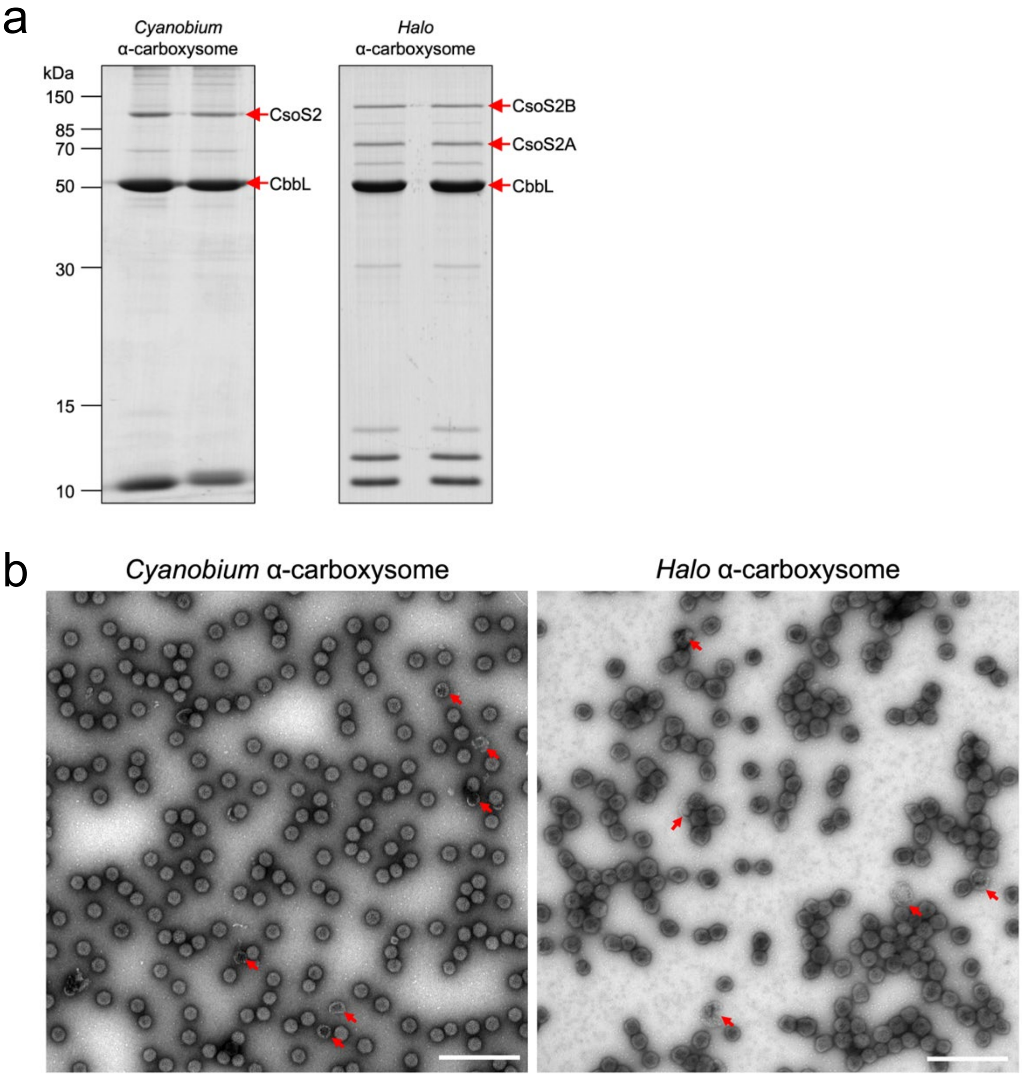

Supplementary Fig. 1 | Preparation of native *Cyanobium* and *Halo* α-carboxysomes. (a) SDS-PAGE of purified *Cyanobium* and *Halo* α-carboxysomes. Red arrows indicate bands of CsoS2, CsoS2A/B, and CbbL. The estimated molar ratio of CsoS2, CsoS2B, and CsoS2A was 1.00 ± 0.24: 0.82 ± 0.03: 1.46 ± 0.13, determined from protein band intensities calibrated to equal CbbL content. (b) Negative-staining EM of purified *Cyanobium* and *Halo* α-carboxysomes. The results show that 98 ± 1% and 96 ± 2% (average ± SD, from three independent batches of isolation) of purified *Cyanobium* and *Halo* carboxysomes are structurally intact (n = 840 and 644 for *Cyanobium* and *Halo* carboxysomes, respectively). Broken carboxysomes were marked by red arrows. Scale bar = 500 nm.

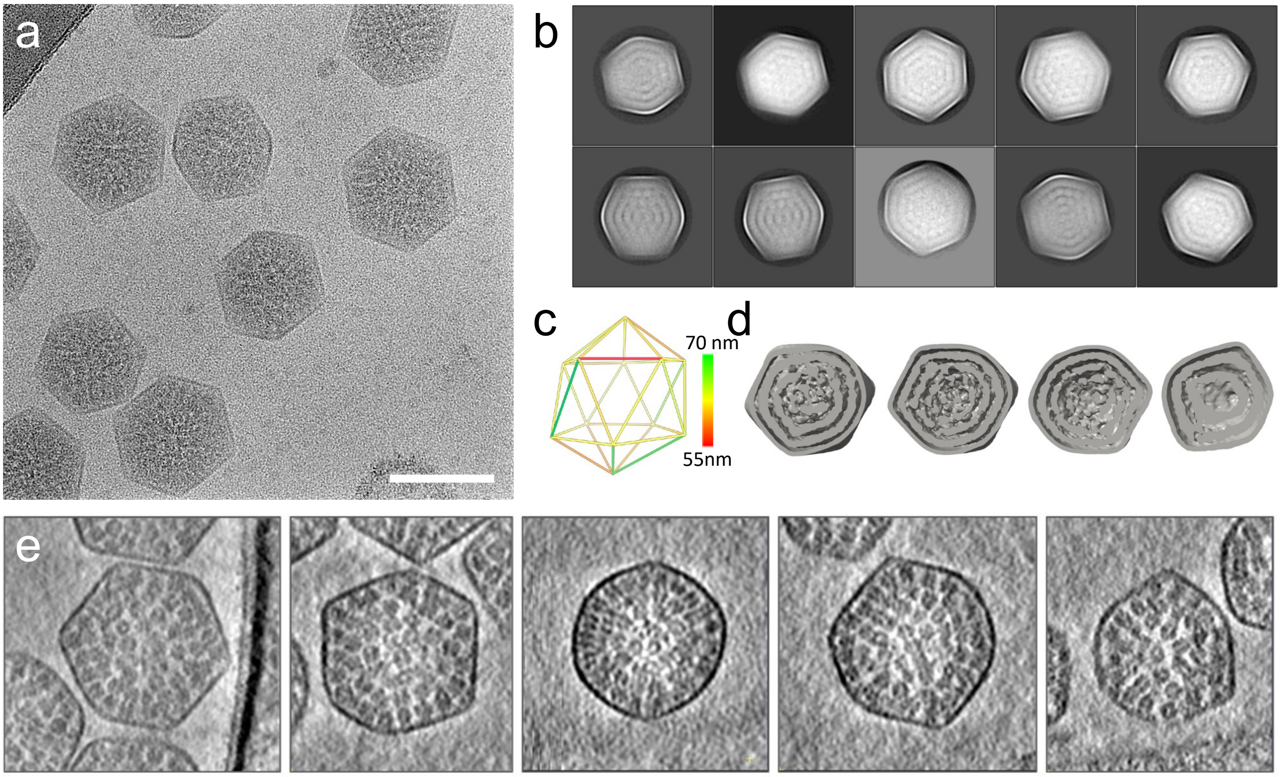

Supplementary Fig. 2 | CryoEM SPA of *Cyanobium* carboxysomes. (a) A representative micrograph of *Cyanobium* carboxysomes (n=13606). (b) 2D class averages of *Cyanobium* carboxysomes. (c-d) Reconstruction of *Cyanobium* carboxysomes without symmetry, shown in geometry (c) and cross-sections (d). The distances between vertices were colored according to their length (55 nm to 70 nm from red to blue). (e) A gallery of non-icosahedral *Cyanobium* carboxysomes (central tomographic slices) with variable size and shape (n=152317). Scale bar 100 nm.

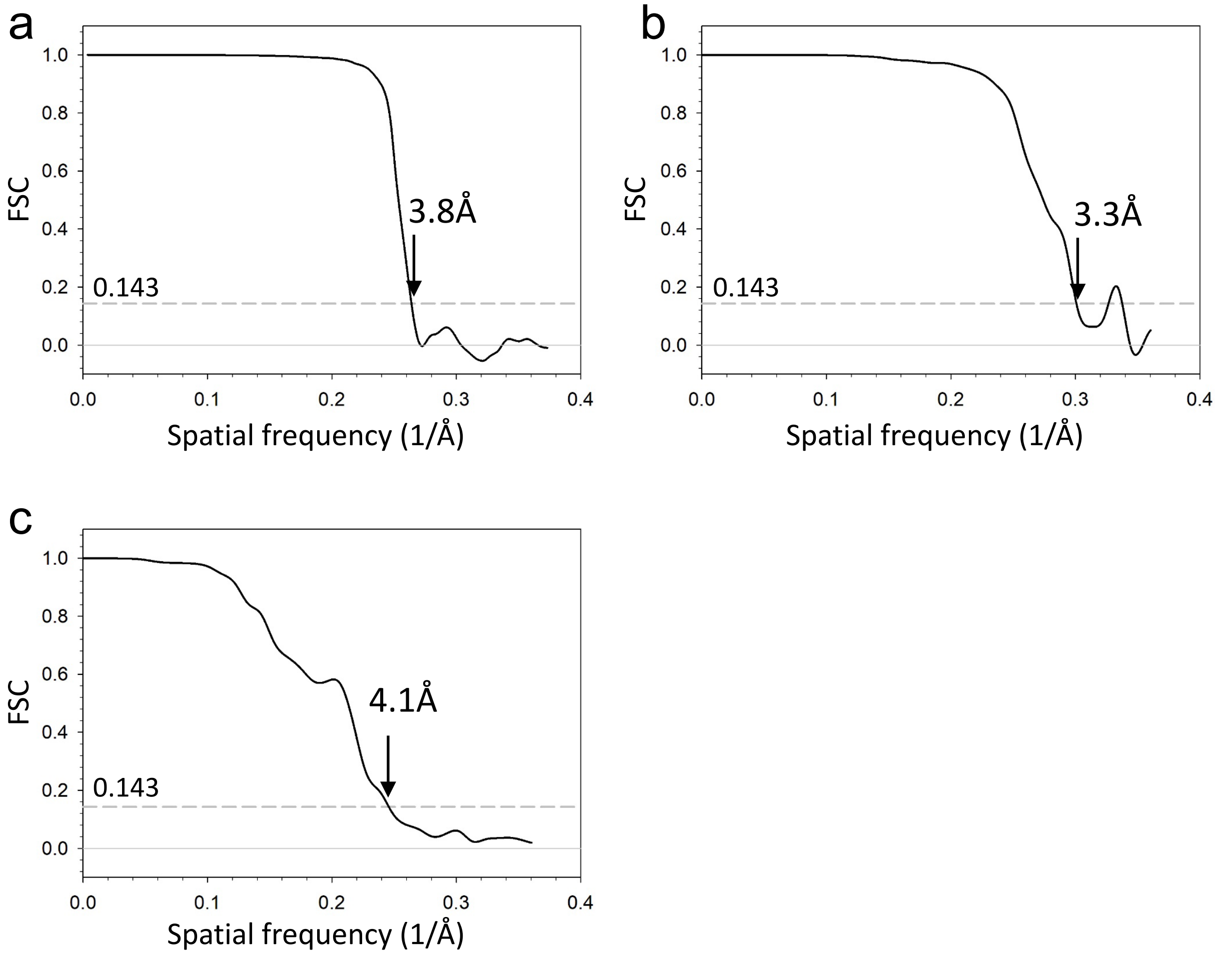

Supplementary Fig. 3 | Fourier Shell Correlation plots of cryoET STA of Rubiscos. (a) FSC of Rubiscos STA map from *Cyanobium* carboxysomes. (b) FSC of Rubisco STA map from *Halo* carboxysomes. (c) FSC of Rubisco dimer STA map from *Halo* carboxysomes. Source data are provided as a Source Data file

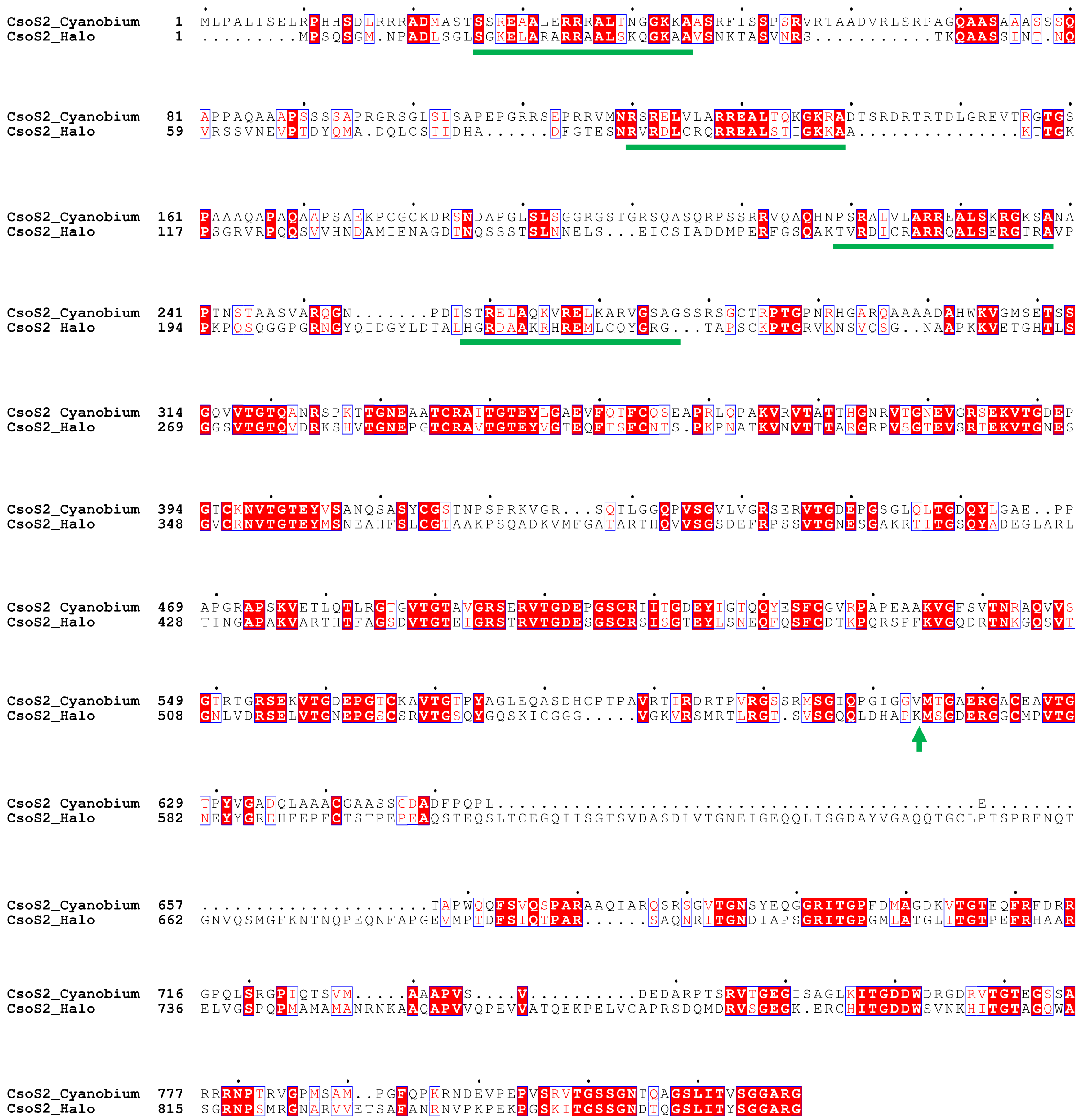

Supplementary Fig. 4 | Sequence alignment of CsoS2 proteins from *Cyanobium* and *Halothiobacillus*. Green lines indicate the potential binding domains with Rubisco. Green arrow indicates the ribosomal frameshift site in *Halo* CsoS2.

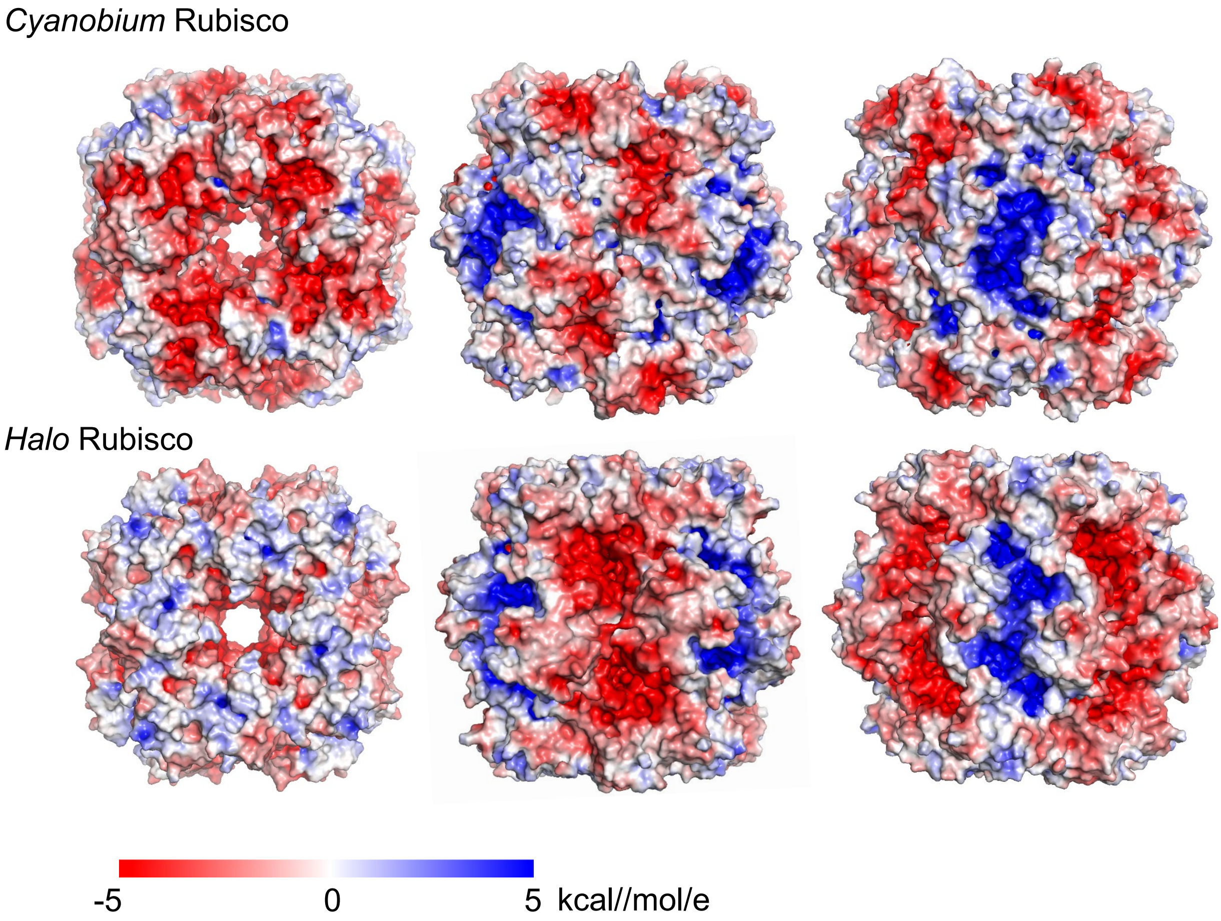

Supplementary Fig. 5 | Surface electrostatic potential of Rubiscos from *Cyanobium* and *Halo*, presented in top view (left, along the 4-fold axis) and two side views (center and right, along the 2-fold axises). The surface electrostatic potential is calculated with APBS plugin in PyMOL. The potentials are on a [−5, 5] red-white-blue color map in units of kcal//mol/e.

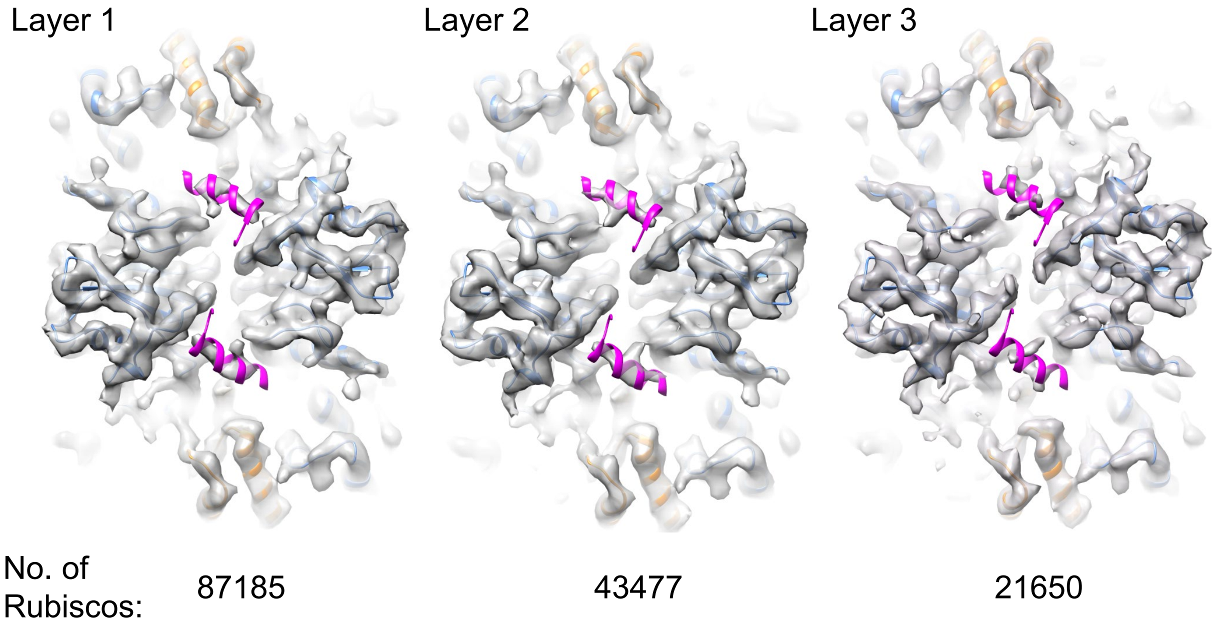

Supplementary Fig. 6 | CsoS2 density in STA maps of Rubiscos in three concentric layers. Rubiscos from three layers were averaged, according to its radial distance distributions in Fig. 2c. From left to right: Layer 1: 350 - 600 Å, layer 2: 250 - 350 Å and layer 3: 0 - 250 Å. Numbers of subtomograms in each layer are listed below.

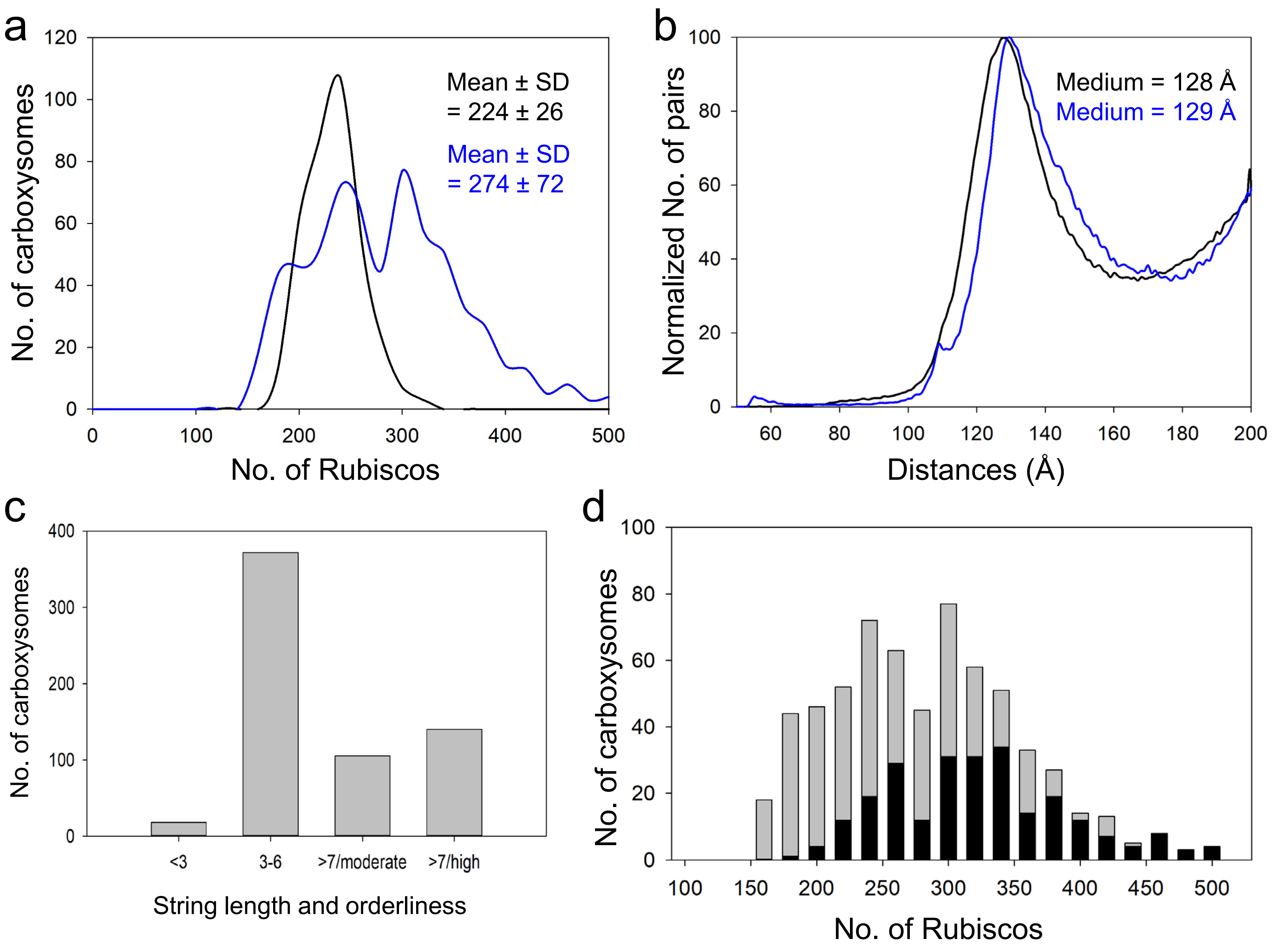

Supplementary Fig. 7 | Comparison of *Cyanobium* and *Halo* carboxysomes. (a) Histogram of Rubisco numbers in *Cyanobium* carboxysomes (black, n=360) and *Halo* carboxysome (blue, n=635). (b) Pairwise Rubisco distances in *Cyanobium* carboxysomes (black, n=1.66 million) and *Halo* carboxysome (blue, n=1.12 million). Distances within 200 Å are plotted. The peak distances are shown. (c) Classification of Rubisco string length (number of Rubiscos in the string) and orderliness in *Halo* carboxysomes (n=635 carboxysomes). (d) Distribution of carboxysomes with (black bar) and without (gray) ordered spiral strings in *Halo* carboxysomes (n=635), plotted as a histogram of Rubisco numbers. Source data are provided as a Source Data file.
