# Supplementary material for: Structure and assembly of cargo Rubisco in two native α-carboxysomes
Source: Nat Commun. 2022 Jul 25;13:4299. doi: 10.1038/s41467-022-32004-w (PMC9314367; doi:10.1038/s41467-022-32004-w)
Supplement: Supplementary file 2 — Description of Additional Supplementary Files [file 41467_2022_32004_MOESM2_ESM.pdf]

File name: Supplementary Movie 1

Description: Sliceview of Cyanobium carboxysomes from tomographic reconstruction. Scalebar 50 nm.

File name: Supplementary Movie 2

Description: Structure of Rubisco from Cyanobium carboxysomes by cryoET subtomogram averaging. Sliceview from the top to bottom of Rubisco. The CbbL and CbbS are colored in blue and gold, respectively, with CsoS2 peptide in magenta.

File name: Supplementary Movie 3

Description: Sliceview of a representative cryoET mapback of Cyanobium carboxysome. The movie slices from the top of the carboxysomes to the bottom. The refined position and coordinate for each Rubiscos were placed back to tomograms in Chimera with *Place Object* plugin. Rubiscos in the three concentric layers are colored from outer layer to inner layer with blue gradient. The carboxysome shell is segmented and colored in gray.

File name: Supplementary Movie 4

Description: Sliceview of Halo carboxysomes from tomographic reconstruction.

File name: Supplementary Movie 5

Description: Template matching result showing the positions of matched Rubiscos in five intact carboxysomes.

File name: Supplementary Movie 6

Description: Structure of Rubisco from Halo carboxysomes by cryoET subtomogram averaging. Sliceview from the top to bottom of Rubisco. The CbbL and CbbS are colored in light sea green and gold, respectively.

File name: Supplementary Movie 7

Description: Mapback of Rubiscos in the spiral array in Halo carboxysomes. The position and orientation of individual Rubisco mapped back to the tomogram of Halo carboxysome, shown as square plate perpendicular to the 4-fold symmetry axis of Rubisco and colored according to the cross-correlation values between individual Rubisco and the STA map.

File name: Supplementary Movie 8

Description: Sliceview of a representative cryoET mapback of Halo carboxysome. The movie slices from the top of the carboxysomes to the bottom and focuses on the Rubiscos in the spiral array later. The refined position and coordinate for each Rubiscos were placed back to tomograms in Chimera with *Place Object* plugin. Rubiscos in the spiral array is colored as gold and outside the spiral array is colored in light blue. The carboxysome shell is segmented and colored in gray.
