# Supplementary material for: Structure and assembly of cargo Rubisco in two native α-carboxysomes
Source: Nat Commun. 2022 Jul 25;13:4299. doi: 10.1038/s41467-022-32004-w (PMC9314367; doi:10.1038/s41467-022-32004-w)
Supplement: Supplementary file 11 — Reporting Summary [file 41467_2022_32004_MOESM11_ESM.pdf]

nature portfolio

Corresponding author(s): Peijun Zhang

Last updated by author(s): 21 June 2022

Reporting Summary

Nature Portfolio wishes to improve the reproducibility of the work that we publish. This form provides structure for consistency and transparency in reporting. For further information on Nature Portfolio policies, see our Editorial Policies and the Editorial Policy Checklist.

Statistics

For all statistical analyses, confirm that the following items are present in the figure legend, table legend, main text, or Methods section.

n/a	Confirmed	
☐	☒	The exact sample size (*n*) for each experimental group/condition, given as a discrete number and unit of measurement
☒	☐	A statement on whether measurements were taken from distinct samples or whether the same sample was measured repeatedly
☒	☐	The statistical test(s) used AND whether they are one- or two-sided<br>*Only common tests should be described solely by name; describe more complex techniques in the Methods section.*
☒	☐	A description of all covariates tested
☐	☒	A description of any assumptions or corrections, such as tests of normality and adjustment for multiple comparisons
☒	☐	A full description of the statistical parameters including central tendency (e.g. means) or other basic estimates (e.g. regression coefficient) AND variation (e.g. standard deviation) or associated estimates of uncertainty (e.g. confidence intervals)
☒	☐	For null hypothesis testing, the test statistic (e.g. *F*, *t*, *r*) with confidence intervals, effect sizes, degrees of freedom and *P* value noted<br>*Give P values as exact values whenever suitable.*
☒	☐	For Bayesian analysis, information on the choice of priors and Markov chain Monte Carlo settings
☒	☐	For hierarchical and complex designs, identification of the appropriate level for tests and full reporting of outcomes
☒	☐	Estimates of effect sizes (e.g. Cohen's *d*, Pearson's *r*), indicating how they were calculated

Our web collection on statistics for biologists contains articles on many of the points above.

Software and code

Policy information about availability of computer code

Data collection	cryoET data were collected using serialEM (v3.8) (referenced in methods), and cryoEM data were collected using EPU.
Data analysis	cryoEM data processing: emClarity (v1.5.0.2 and v1.5.3.11); IMOD (v4.9.12) (available and referenced in methods).<br>Model building: AlphaFold2, Coot (WinCoot 0.9.9.1), Chimera (v1.16), Phenix (Phenix v1.20), Namdinator (available and referenced in methods).

For manuscripts utilizing custom algorithms or software that are central to the research but not yet described in published literature, software must be made available to editors and reviewers. We strongly encourage code deposition in a community repository (e.g. GitHub). See the Nature Portfolio guidelines for submitting code & software for further information.

Data

Policy information about availability of data

All manuscripts must include a data availability statement. This statement should provide the following information, where applicable:

- Accession codes, unique identifiers, or web links for publicly available datasets
- A description of any restrictions on data availability
- For clinical datasets or third party data, please ensure that the statement adheres to our policy

All data needed to evaluate the conclusions in the paper are present in the paper and/or the Supplementary information. The cryoET subtomogram averaging density maps and corresponding atomic models have been deposited in the EMDB and PDB, respectively. The accession codes are listed as follows: Cyanobium Rubisco from all the carboxysomal Rubisco (PDB: 7ZC1 and EMD-14617), Cyanobium Rubisco from the outer layer (EMD-14625), middle layer (EMD-14624) inner layer (EMD-14623); Halo Rubisco inside carboxysomes (PDB: 7ZBT and EMD-14590), close to the shell (EMD-14592), within 300 Å from the carboxysome center (EMD-14593) and within the spiral array (EMD-14589). Source data are provided with this paper. Validation reports are provided.

Field-specific reporting

Please select the one below that is the best fit for your research. If you are not sure, read the appropriate sections before making your selection.

☒ Life sciences ☐ Behavioural & social sciences ☐ Ecological, evolutionary & environmental sciences

For a reference copy of the document with all sections, see nature.com/documents/nr-reporting-summary-flat.pdf

Life sciences study design

All studies must disclose on these points even when the disclosure is negative.

Sample size	For cryoET subtomogram averaging, sample sizes were those required for the resolution. The details of datasets, including sample sizes, are listed in Supplementary Table 1.<br>For carboxysome inspection by TEM and SDS-PAGE, sample sizes were not predetermined based on statistical methods, but were determined by according to the standard of the field (at least three independent biological replicates for each sample), which generate sufficient statistics for analysis.
Data exclusions	For cryoET subtomogram averaging data processing, data from the first 60 tilt-series (60/ 157) in the Halo carboxysomes were processed. All the tilt-series from the Cyanobium dataset were processed. A few miss-aligned tilt-series were excluded for further data processing.<br>To quantify the number of Rubiscos in each carboxysome, only the intact carboxysomes were included. Visual inspections on the template-based picked Rubiscos were performed.<br>For SDS-PAGE quantification analysis batch samples with significant lower purity was removed from the analysis to avoid errors caused by other protein contaminants.
Replication	Fro cryoET subtomogram averaging, two randomly divided half datasets were processed independently and combined to give rise to the final structures. The resolution of the structure is assessed by comparing the two independent maps.<br>For carboxysome inspection by TEM and SDS-PAGE, at least three independent biological replicates for each sample were analyzed.
Randomization	The subtomograms were randomly divided into ODD and EVEN datasets, as standard approach implemented in emClarity.
Blinding	Quantification of Rubiscos and identification of Rubisco strings were not blinded as the intact carboxysomes are identified visually and included for quantification.

Reporting for specific materials, systems and methods

We require information from authors about some types of materials, experimental systems and methods used in many studies. Here, indicate whether each material, system or method listed is relevant to your study. If you are not sure if a list item applies to your research, read the appropriate section before selecting a response.

Materials & experimental systems

n/a	Involved in the study
☒	☐ Antibodies
☒	☐ Eukaryotic cell lines
☒	☐ Palaeontology and archaeology
☒	☐ Animals and other organisms
☒	☐ Human research participants
☒	☐ Clinical data
☒	☐ Dual use research of concern

Methods

n/a	Involved in the study
☒	☐ ChIP-seq
☒	☐ Flow cytometry
☒	☐ MRI-based neuroimaging
